# Insulin Fused to Apolipoprotein A-I Reduces Body Weight and Steatosis in DB/DB Mice

**DOI:** 10.3389/fphar.2020.591293

**Published:** 2021-02-19

**Authors:** Nuria Ardaiz, Celia Gomar, Marcos Vasquez, Shirley Tenesaca, Myriam Fernandez-Sendin, Claudia Augusta Di Trani, Virginia Belsué, Javier Escalada, Ulrich Werner, Norbert Tennagels, Pedro Berraondo

**Affiliations:** ^1^ Program of Immunology and Immunotherapy, Cima Universidad de Navarra, Pamplona, Spain; ^2^ IdiSNA, Navarra Institute for Health Research, Pamplona, Spain; ^3^ Department of Endocrinology, Clínica Universidad de Navarra, Pamplona, Spain; ^4^ Centro de Investigación Biomédica en Red de la Fisiopatología de la Obesidad y Nutrición (CIBEROBN), Madrid, Spain; ^5^ Sanofi-Aventis Deutschland GmbH, TA Diabetes, Frankfurt am Main, Germany; ^6^ Centro de Investigación Biomédica en Red de Cáncer (CIBERONC), Madrid, Spain

**Keywords:** fusion protein, gene therapy, liver-targeted insulin, non-alcoholic fatty liver disease, apolipoprotein A-I

## Abstract

**Background:** Targeting long-lasting insulins to the liver may improve metabolic alterations that are not corrected with current insulin replacement therapies. However, insulin is only able to promote lipogenesis but not to block gluconeogenesis in the insulin-resistant liver, exacerbating liver steatosis associated with diabetes.

**Methods:** In order to overcome this limitation, we fused a single-chain insulin to apolipoprotein A-I, and we evaluated the pharmacokinetics and pharmacodynamics of this novel fusion protein in wild type mice and in db/db mice using both recombinant proteins and recombinant adenoassociated virus (AAV).

**Results:** Here, we report that the fusion protein between single-chain insulin and apolipoprotein A-I prolonged the insulin half-life in circulation, and accumulated in the liver. We analyzed the long-term effect of these insulin fused to apolipoprotein A-I or insulin fused to albumin using AAVs in the db/db mouse model of diabetes, obesity, and liver steatosis. While AAV encoding insulin fused to albumin exacerbated liver steatosis in several mice, AAV encoding insulin fused to apolipoprotein A-I reduced liver steatosis. These results were confirmed upon daily subcutaneous administration of the recombinant insulin-apolipoprotein A-I fusion protein for six weeks. The reduced liver steatosis was associated with reduced body weight in mice treated with insulin fused to apolipoprotein A-I. Recombinant apolipoprotein A-I alone significantly reduces body weight and liver weight, indicating that the apolipoprotein A-I moiety is the main driver of these effects.

**Conclusion:** The fusion protein of insulin and apolipoprotein A-I could be a promising insulin derivative for the treatment of diabetic patients with associated fatty liver disease.

## Introduction

The therapeutic goal of the management of type 1 and type 2 diabetic patients is an optimal glucose regulation to reduce the risk for complications such as retinopathy, nephropathy, and neuropathy. Insulin modifications with differences in their time of onset and duration of action are available for glucose regulation in diabetic patients. Whereas modifications resulting in a prolonged pharmacokinetic profile are used to mimic the basal insulin supply, rapid-acting insulins are administered for prandial control (King, 2012).

However, in any case, supraphysiological concentrations of insulin have to be injected subcutaneously as the exogenous supply does not replicate the normal physiological delivery of insulin into the portal circulation, which results in higher exposure of the liver than the peripheral tissues such as fat and muscle (Chap et al., 1987). Although subcutaneous administration of insulin is able to control glycemia in patients, it consequently exerts a variety of metabolic abnormalities, including excessive glycemic fluctuations, dyslipidemia, and a reduction in plasma IGF-1 coupled to higher levels of growth hormone. These alterations may contribute to the long-term micro- and macrovascular complications of diabetes (Sonksen et al., 1993). Thus, novel insulin formulations are needed to overcome these limitations.

Liver is a key insulin target organ both in healthy people and in the insulin-resistant type 2 diabetic patient. Hepatic glucose production is a major contributor to the hyperglycemia characteristic of type 2 diabetes. The increased glucose release from the liver to the blood is the combination of gluconeogenesis and glycogenolysis (Hatting et al., 2018). Physiological hyperinsulinemia completely blocks glycogenolysis and partially inhibits gluconeogenesis (Gastaldelli et al., 2001). In type 2 diabetic patients, glycogenolysis does not contribute to the hepatic glucose production and insulin loses its ability to reduce gluconeogenesis (Magnusson et al., 1992). In contrast, in these type 2 diabetic patients, insulin retains its ability to enhance lipogenesis in the liver, creating a negative feedback that sustains the metabolic alterations present in diabetes mellitus (Li et al., 2009). Indeed, non-alcoholic fatty liver disease (NAFLD) is commonly associated with insulin resistance and obesity (Marchesini et al., 1999). Thus, refinement of the pharmacokinetic/pharmacodynamic profile of insulin analogs is required to improve the insulin therapy of patients resulting in better outcomes and reduced side effects (Mazzola, 2012). Currently, insulin can be delivered into the portal vein only by intraperitoneal insulin pumps (Ruotolo et al., 1990), by pancreatic transplantation with enteric drainage, or by islet cell transplantation (Stratta et al., 1995). At present, all these methods have significant drawbacks that preclude their use as treatment for a vast majority of insulin users. A potential solution to this problem is the development of insulin analogs that have a greater effect on the liver than at the periphery. Several attempts using fusion-protein technologies have been made to target the liver including thyroxine (thyroxyl-insulin), pegylation, or fusing proinsulin to transferrin (Shojaee-Moradie et al., 2000; Wang et al., 2014; Jacober et al., 2016). A potential limitation of these approaches is that the accumulation of triglycerides in the insulin-resistant liver induces a vicious circle that ends up in a fatty liver that is resistant to the beneficial activity of insulin but sensitive to the lipogenic activity of insulin (Marchesini et al., 1999; Li et al., 2009).

To design an improved liver-targeted insulin, we focused on apolipoprotein A-I as a molecular scaffold. Apolipoprotein A-I has a natural tropism for the liver (Kim et al., 2007; Ding et al., 2012) and a long half-life in circulation. A clinical trial using *in vitro* reconstituted HDLs reported an elimination half-life between 19.3 and 92.8 h (Easton et al., 2014). In addition, HDL may control glucose homeostasis through mechanisms including insulin secretion, direct glucose uptake by muscle via the AMP-activated protein kinase, and enhanced insulin sensitivity (Drew et al., 2012; Wu et al., 2019). We report here that the fusion of single-chain insulin to apolipoprotein A-I generates a novel basal insulin that is accumulated in the liver and reduces body weight and hepatic steatosis. Therefore, it could be beneficial for the treatment of diabetic patients with a concomitant NAFLD.

## Material and Methods

### Cells and Reagents

The human hepatocyte cell line IHH was provided to us by Dr. Bart Staels (Samanez et al., 2012). Cells were cultured in Williams E medium (Invitrogen, CergyPontoise, France), containing 11 mM glucose and supplemented with 10% fetal calf serum (FCS) (Invitrogen), 100 μg/ml penicillin, 100 μg/ml streptomycin, 20 mU/ml bovine insulin (Sigma-Aldrich, St. Quentin Fallavier, France) and 50 nM dexamethasone (Sigma-Aldrich). Murine 3T3-L1 fibroblasts were provided by Dr Matilde Bustos (CIMA, Pamplona, Spain). Cells were maintained in DMEM supplemented with 10% FCS, 100 μg/ml penicillin, 100 μg/ml streptomycin. L6-GLUT4-myc cells (Baus et al., 2008) were grown in MEMα supplemented with 10% FCS, 2 μg/ml blasticidin (Calbiochem, San Diego, CA), 0.5 μg/ml puromycin (InvivoGen, San Diego, CA), 200 μg/ml hygromycin (Invitrogen). Chinese hamster ovary cells overexpressing the A isoform of the human IR (CHO-hIR) were maintained in DMEM (Gibco, Grand Island, NY) with 5.5 mM glucose (1,000 mg/L) supplemented with 10% FCS, 1% penicillin/streptomycin (Gibco), 100 µM MEM non-essential amino acids (Gibco) and 100 µM methotrexate (Sigma, St. Louis, MO). HepG2 cells were grown in DMEM supplemented with 10% FCS, 100 μg/ml penicillin, and 100 μg/ml streptomycin.

Insulin glargine (Lantus) was from Sanofi-Aventis (Paris, France). The recombinant fusion proteins insulin fused to apolipoprotein A-I (Insulin-Apo), apolipoprotein A-I (Apo) and insulin fused to albumin (Albulin) were expressed in *E. coli* and purified by GenScript Corp. (New Jersey, United StatesUnited States). Insulin was purchased from GERBU Biotechnik (Heidelberg, Germany).

### *In Vitro* Insulin Activity Assays

Murine 3T3-L1 fibroblasts were grown and differentiated into adipocytes in a 24-well plate containing 2 × 10^4^ 3T3-L1 adipocytes. 24 h before the experiment, growth medium was replaced with DMEM (Life Technologies, Ghent, Belgium) without fetal bovine serum. Then, cells were washed twice with KRB medium, and insulin and Insulin-Apo were added for 2 h, followed by addition of 0.5 µCi 2-deoxy-d-[3H]glucose for 5 min at 37°C. Incorporated 3H was quantified after extensive washing with KRB medium.

L6-GLUT4-myc cells were plated in 96 well Cytostar-T scintillating microplates (Amersham, Freiburg, Germany) at 3.0 × 10^4^ cells per well. After 48 h, cells were serum-starved (3–4 h) and treated with insulin or Insulin-Apo as indicated. To analyze glucose uptake, cells were washed twice with KRB and incubated for 25 min with insulin or Insulin-Apo. [14C] labeled 2-deoxyglucose (0.01 MBq per well, Amersham) was added, and cells were incubated for another 25 min. Non-specific uptake was determined in the presence of 40 µM cytochalasin B (Calbiochem, Los Angeles, CA). This value was subtracted from all other values. Radioactive counts were measured in a Wallac Microbeta counter (Perkin Elmer, Foster City, CA). Uptake of 2-deoxyglucose is detected as counts per minute (cpm) and presented as percentage of maximum uptake induced by insulin.

Chinese hamster ovary cells expressing human IR were used for IR autophosphorylation assay and HepG2 for the AKT phosphorylation assay using In-Cell Western technology as previously described (Sommerfeld et al., 2010).

8 × 10^5^ IHH cells were incubated in 6-well plates in DMEM with 100 U/ml penicillin, 100 μg/ml streptomycin, and 0.1% bovine serum albumin (BSA) (Sigma-Aldrich). After o/n incubation, cells were treated for 15 min with various concentrations of insulin or insulin-Apo. Cells were collected in loading buffer [1 M Tris-HCL (pH 6.8), 20% SDS, 0.05% bromophenol blue and 50% glycerol]. Proteins were separated with 8% SDS-PAGE, transferred to nitrocellulose membranes and stained with Ponceau red solution (Sigma-Aldrich) to verify equal loading of proteins. Membranes were then blocked for 2 h in TBS-Tween-20 [50 mM Tris-HCL (pH 7.6), 200 mM NaCl and 0.05% Tween-20] with 5% BSA (Sigma-Aldrich) and then primary antibody against phospho-AKT (Ser473) and AKT (Cell Signaling Technologies, Beverly, MA) was added and incubated overnight at 4°C. After further washings, membranes were incubated for 40 min with horseradish peroxidase-conjugated secondary antibody (Santa Cruz Biotechnology, Santa Cruz, CA). The immunoreactive proteins were detected with enhanced chemiluminescence (Amersham Biosciences).

### Animal Experiments

Seven weeks-old female C57BL/6J mice or BKS.Cg- + Lepr^db^/+ Lepr^db^/OlaHsd mice were purchased from Harlan Laboratories (Barcelona, Spain). For experiments with AAV vectors, female db/db mice from a colony maintained at the University of Navarra were used. Mice were kept in the University of Navarra animal facilities and cared for according to the institutional guidelines for animal care.

In the experiments with AAV vectors, female 10 weeks-old db/db mice were treated with 5 × 10^12^ viral genomes AAV encoding luciferase (AAVLuc), AAV encoding Insulin-Apo (AAVInsulin-Apo) or AAV encoding albulin (AAVAlbulin) (N = 8 per group). In the experiments with repeated administration of the recombinant protein, male 10 weeks-old db/db mice were treated daily for 6 weeks with 5 U/kg human insulin s.c., 26 nmol/kg apolipoprotein A-I s.c., or 26 nmol/kg Insulin-Apo s.c. Bodyweight was measured once per week, and food intake twice per week for three months, and mice were sacrificed at the end of the study. Serum was obtained and stored at –20°C until assayed. Epididymal and inguinal fat were surgically removed as representatives of visceral and subcutaneous fat, respectively. Fatty deposits and livers were weighed, and liver samples were stored in formalin or frozen at‐80°C. The number of mice used in the experiment depicted in Figures 6, 7 are n = 13 for PBS and Insulin-Apo groups and n = 8 for the insulin-treated group. The number of mice of the experiment depicted in Figure 8 are n = 6 for PBS group, n = 12 for Insulin-Apo-treated group and n = 5 for Apo-treated group. For induction of diabetes, C57BL/6J wild-type mice were given a single intravenous dose of 175 mg/kg of streptozotocin (Sigma-Aldrich). 4 days later, animals were treated with insulin or Insulin-Apo intravenously (139 nmol/kg), and blood glucose was monitored before and sequentially after treatments.

### ELISAs

The serum concentration of insulin after intravenous administration of 55 nmol/kg of insulin or Insulin-Apo was determined in serum samples obtained at several time points and stored at -20°C. A commercial ELISA kit for the detection of human insulin (RayBiotech, Norcross, GA) was used according to manufacturer's instructions.

### Adeno-Associated Virus

All AAV plasmids contain an expression cassette flanked by two inverted terminal repeats from the AAV2. The expression cassette contains the ubiquitous human 1-α elongation factor (EF1-α) promoter, the gene encoding for luciferase or the cDNA encoding for Insulin-Apo or insulin fused to albumin (albulin). Optimized sequences for production in *E. coli* were used to reduce the protein production per cell and to avoid toxicity due to hyperinsulinemia. AAV2/8 vectors were produced by polyethyleneimine transfection of 293T cells. The resulting viral vectors (AAVLuc, AAVInsulin-Apo, or AAVAlbulin) were harvested and purified by iodixanol-gradient centrifugation, followed by filtration and further concentration against phosphate-buffered saline–5% saccharose. Viral titers in terms of genome copies per milliliter (gc/ml) were determined by quantitative real-time polymerase chain reaction (RT-PCR) (Fernandez-Sendin et al., 2020).

### Microarray Analysis

Six hours after s.c. administration of saline, Insulin-Apo or albulin (55 nmol/kg) to C57BL/6J mice, liver samples were isolated and RNA extracted using Maxwell 16 LEV simplyRNA purification kit (Promega Madison, WI). Samples were then processed following Affymetrix recommendations, and cRNA was hybridized to the Affymetrix Mouse Gene 1.0 ST array. Both background correction and normalization were done using the Robust Multichip average algorithm (Irizarry et al., 2003). Then, a filtering process was performed to eliminate probe sets with low expression levels. Applying the criterion of an expression value greater than 5 in at least 2 samples of one of the experimental conditions, 258024 probe sets were selected for the statistical analysis. R and Bioconductor were used for preprocessing and statistical analysis(Gentleman, 2005). LIMMA (Linear Models for Microarray Data) was used to find which probe sets showed significant differential expression between experimental conditions (Smyth, 2004). Genes affected by Insulin-Apo, albulin treatments were identified as significant based on a B statistic cutoff (B > 0).

Functional enrichment analysis of Gene Ontology (GO) categories was carried out using a standard hypergeometric test (Draghici, 2003). The biological knowledge extraction was complemented by the use of Ingenuity Pathway Analysis (Ingenuity Systems, www.ingenuity.com), which database includes manually curated and fully traceable data derived from literature sources. Microarray data are available on the Gene Expression Omnibus (GEO) website (accession number: GSE160105).

### Quantitative RT-PCR

Total RNA from mice livers was isolated using Maxwell 16 LEV simplyRNA purification kit (Promega). RNA was treated with DNase I and retrotranscribed to cDNA with MMLV RT in the presence of RNase OUT (all reagents from Invitrogen, Carlsbad, CA, United States) according to manufacturer’s instructions. Primers for quantitative RT-PCR are listed in Supplementary Table S1. RPLP0 was used to standardize gene expression. The mRNA values were represented by formula 2^ΔCt^, where ΔCt indicates the difference in the threshold cycle between RPLP0 and the target genes (all reagents from BioRad, Hercules, CA).

### Serum Biochemistry

AST, ALT, and total cholesterol serum measurements were done using a Cobas Mira Autoanalyzer (Roche Diagnostic, Basel, Switzerland). Glycemia was determined using Accu-Chek Aviva (Roche Diagnostic, Barcelona, Spain).

### Histology

Sections of paraffin-embedded tissues were stained with H&E. For hepatic lipid accumulation analyses, optimal cutting temperature compound (Sakura, Torrance, CA)-embedded frozen livers were sectioned at 10 μm and stained with Oil Red O (Sigma, St. Louis, MO) according to a standard protocol. Representative images were captured with an Axio Observer Z1 (Carl Zeiss, Jena, Germany). Automated quantification was performed using the ColorThershold function of Matlab 7.1.0.83 R14 (Mathworks, Natick, Mass). Background areas were determined in H&E-stained tissue slides and red areas were determined for Oil Red O-stained tissue slides.

### Statistical Analysis

All statistical analyses were performed with Prism software (GraphPad Software 8.4.3, Inc.). Time series were fitted to a model, and treatments were compared using the extra sum-of-squares F test. Differences among three or more groups were analyzed by one-way ANOVA, followed by Sidak's posttest. *p* values < 0.05 were considered to be statistically significant.

## Results

### *In Vitro* Activity of Single-Chain Insulin Fused to Apolipoprotein A-I

To construct an insulin derivative fused to apolipoprotein A-I, we selected a single-chain insulin described previously (Rajpal et al., 2009). This single-chain insulin was fused to the N-terminus of human apolipoprotein A-I with a short linker GAP. To facilitate the purification step, a His-tag was added to the C-terminus end of the apolipoprotein A-I (Supplementary Figure S1A).

**FIGURE 1:**
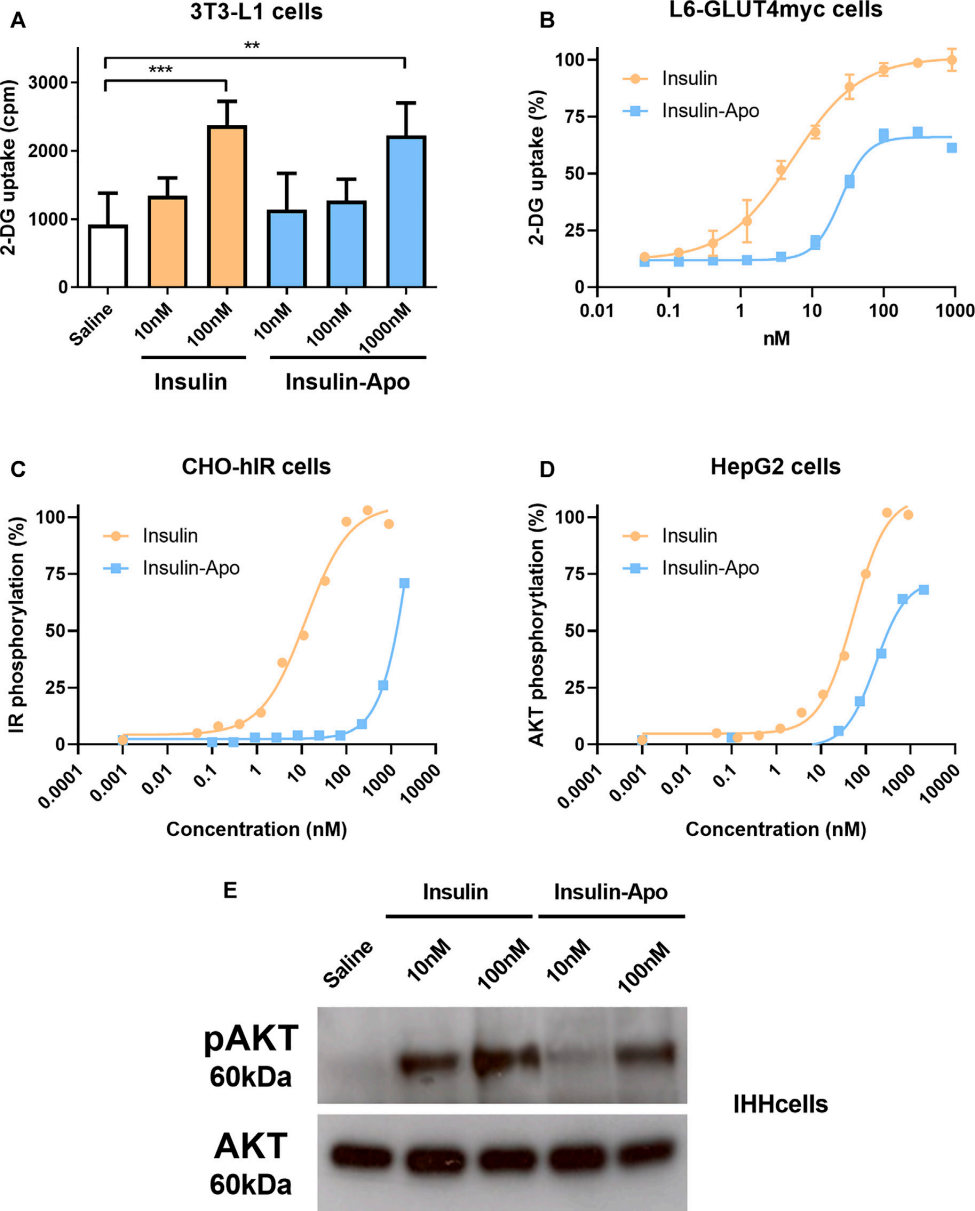
*In vitro* activity of Insulin-Apo. Insulin and Insulin-Apo effects on **(A)** 2-deoxyglucose uptake in 3T3-L1 adipocytes (n = 4) or **(B)** L6-GLUT4myc cells (n = 4); **(C)** phosphorylation of the insulin receptor in CHO-hIR cells; **(D)** phosphorylation of AKT in HepG2 cells. **(E)** Western blot of phospho-AKT signaling in hepatic IHH cells. **p < 0.01, ***p < 0.001.

The activity of the recombinant fusion protein was evaluated, analyzing the uptake of deoxyglucose by 3T3-L1 adipocytes. A concentration of 100 nM of insulin achieved a significant increase in the glucose uptake, while 10-fold higher concentration of Insulin-Apo was required to obtain a similar increase (Figure 1A). This result was confirmed in L6-GLUT4 myc myocytes. The EC50 for insulin was 5.3 nM, while the EC50 with the fusion protein was reached at 25.2 nM (Figure 1B). To further analyze the *in vitro* activity of the fusion protein, the autophosphorylation of the insulin receptor (IR) was determined in CHO cells transfected with the human IR (CHO-hIR). A dose-dependent effect was observed both with human insulin and with Insulin-Apo. In line with previous results, the EC50 was reached at a lower concentration of insulin (12.3 nM) than Insulin-Apo (4.3 µM) (Figure 1C). Finally, we evaluated the activity of human insulin and insulin fused to apolipoprotein A-I in hepatic cell lines. Phosphorylation of AKT is an important signaling event triggered upon insulin binding to its receptor. We observed clear phosphorylation of AKT upon insulin or Insulin-Apo treatment in both Hep2G cells (Figure 1D) and IHH cells (Figure 1E), that maintain features of differentiated human hepatocytes (Samanez et al., 2012). However, recombinant Insulin-Apo required approximately 10-fold higher concentration than insulin to phosphorylate AKT (Figures 1D,E).

### Pharmacokinetic and Pharmacodynamic Properties After Intravenous Administration

**FIGURE 2:**
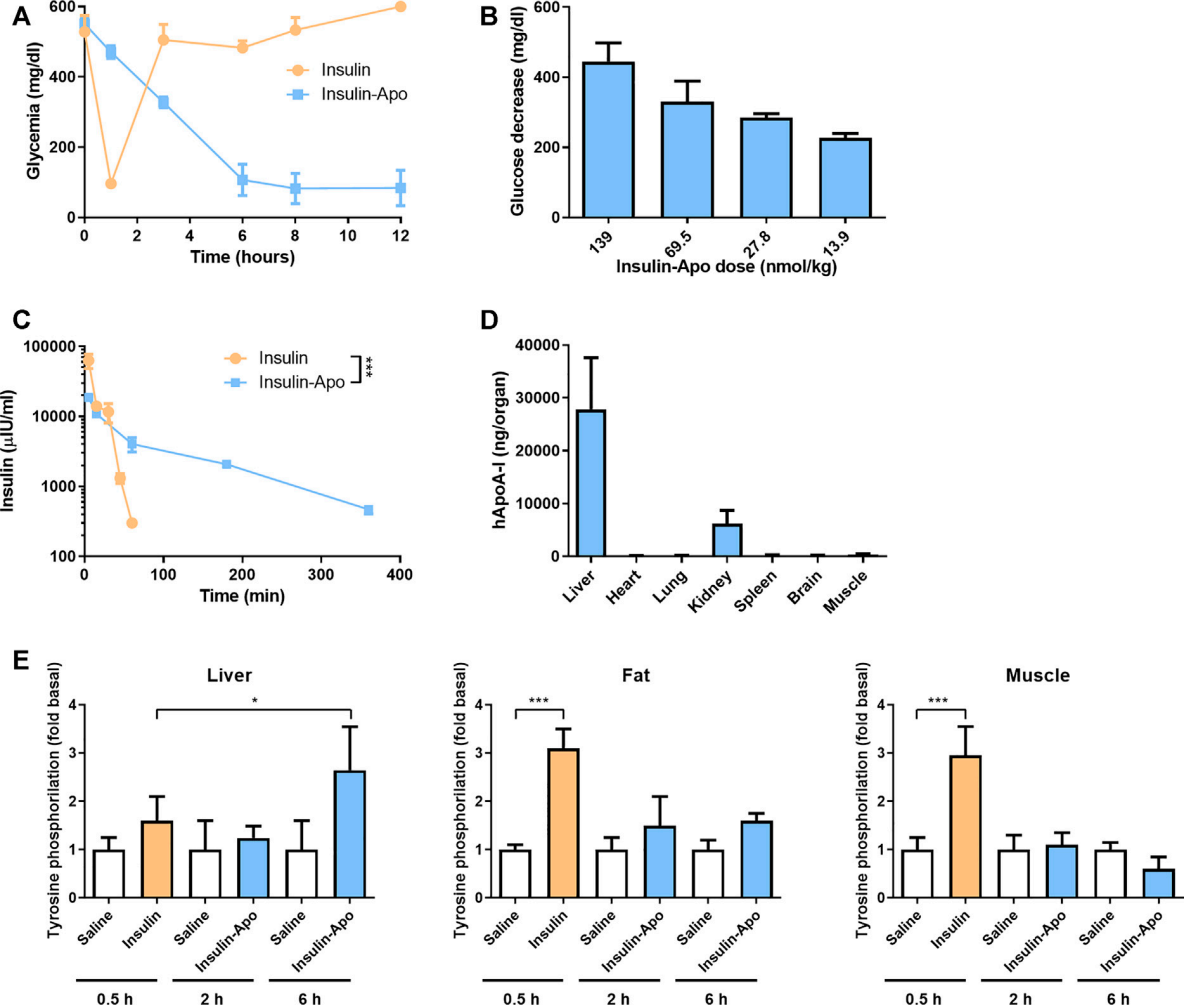
Insulin-Apo pharmacokinetics/pharmacodynamics after intravenous administration. **(A)** Streptozotocin-induced diabetic C57BL/6J mice received 139 nmol/kg insulin or Insulin-Apo i.v. At various time points, mice were bled, and glycemia was determined with a glucometer (n = 3). **(B)** Reduction in glycemia 6 h after administration of different doses of Insulin-Apo i.v. to streptozotocin-induced diabetic C57BL/6J mice. **(C)** C57BL/6J mice received 55 nmol/kg of insulin or, Insulin-Apo **i**.v. At various time points, mice were bled and samples assayed by ELISA for the presence of human serum insulin (n = 3). ***p < 0.001. **(D)** To analyze biodistribution, C57BL/6J mice received 110 nmol/kg Insulin-Apo **i**.v. Three hours after administration, the animals were sacrificed, and several organs were removed. The amount of human apoA-I in the organ homogenates was determined by ELISA (n = 4). **(E)** Tyrosine phosphorylation of the insulin receptor in liver, fat tissue, and muscle was determined by Western blot of tissue samples from C57BL/6J mice 0.5 h after treatment with 1 U/kg s.c. human insulin and 2 and 6 h after treatment with 200 nmol/kg s.c. Insulin-Apo (n = 3).

To analyze the *in vivo* activity of the recombinant protein Insulin-Apo, C57BL/6J mice were treated with 175 mg/kg streptozotocin intravenously. Four days later, induction of diabetes was determined by analyzing the glycemia. Mice with glycemia over 500 mg/dl were included in the experiments. First, a dose of 139 nmol/kg Insulin-Apo was administered intravenously, and glycemia was determined at 0, 1, 3, 6, 8, and 12 h after drug administration. The glucose levels decreased slowly, reaching a plateau at 6 h after injection (Figure 2A). The glycemia remained low, and finally, mice were sacrificed due to the profound hypoglycemia. In contrast, insulin promoted a sharp decrease in glucose levels 1 h after administration but returned to baseline values after 3 h (Figure 2A). Then, a dose-finding study was performed administering different doses of Insulin-Apo to streptozotocin-induced diabetic mice and analyzing the glycemia 6 h after drug administration. A clear dose-response was obtained (Figure 2B), and an intermediate dose of 55 nmol/kg was selected for further studies.

Next, we performed experiments to determine the plasmatic half-life of the protein. C57BL/6J mice received 55 nmol/kg insulin or Insulin-Apo intravenously. At several time points, mice were bled, and samples were assayed with a human insulin ELISA. Insulin plasmatic levels decayed quickly after administration of regular insulin, and 60 min after intravenous injections, no more insulin was detectable. The estimated half-life was 9 min. In sharp contrast, the plasmatic levels of Insulin-Apo decreased slowly with an estimated elimination half-life of 109 min (Figure 2C).

Finally, we performed experiments to analyze the liver targeting of Insulin-Apo. Mice were injected intravenously with Insulin-Apo, and 3 h later, the presence of human apolipoprotein A-I in different organs was detected by ELISA. A signal over the background could be detected in all the organs analyzed. The highest amount of Insulin-Apo was detected in the liver (Figure 2D), reflecting the accumulation of apolipoprotein A-I in the liver before degradation. We also detected high amounts of the recombinant protein in kidneys. This is consistent with other reports describing that the second elimination mechanism for apolipoprotein A-I is renal clearance (Kim et al., 2007). The tyrosine phosphorylation of the insulin receptor was analyzed in liver, fat, and muscle tissue 0.5 h after insulin administration and 2 and 6 h after Insulin-Apo administration. These time points were selected based on the maximum glycemia reduction. The phosphorylation of the insulin receptor increased significantly in fat and muscle but not in liver. In contrast, Insulin-apo induced the insulin receptor phosphorylation only at the liver at 6 h but not at 2 h, indicating a progressive accumulation in the liver (Figure 2E).

### Differential Activity in Liver

**FIGURE 3:**
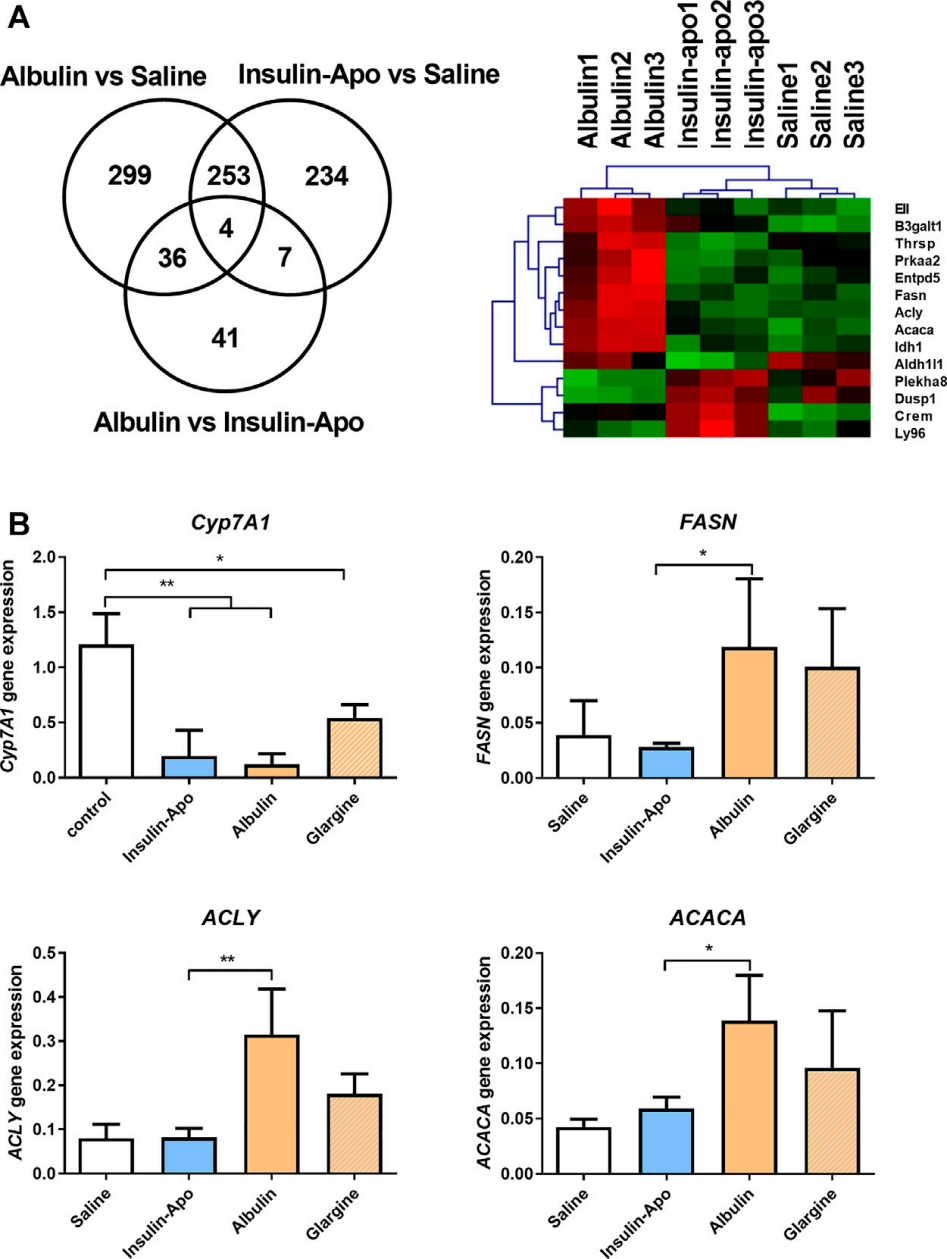
Differential effects on gene expression in the liver. **(A)** Venn diagram and heatmap of the analysis of the modulation of gene expression in the liver by the different insulin derivatives. C57BL/6J mice received s.c. saline, or 55 nmol/kg Albulin or Insulin-Apo. Six hours after administration, the animals were sacrificed, and the livers were extracted. RNA was isolated, and samples were studied using microarray expression (n = 3). **(B)** C57BL/6J mice received subcutaneously saline or 110 nmol/kg Insulin-Apo, or Albulin. Six hours after administration, the animals were sacrificed, and the liver was extracted. RNA was isolated, and RT-PCR was performed. Abbreviations: Cyp7A1, cholesterol 7 alpha-hydroxylase; ACACA, acetyl-CoA carboxylase; ACLY, ATP citrate lyase; and FASN, fatty acid synthase (n = 3). *p < 0.05, **p < 0.01, ***p < 0.001.

In order to further evaluate the *in vivo* properties of the new insulin derivative, we compared the pharmacodynamic properties in the liver to other insulin derivatives with long half-life in circulation: the fusion protein of albumin and insulin (Albulin) (Duttaroy et al., 2005). We administered subcutaneously saline, Albulin or Insulin-Apo to wild type mice, and 6 h later, livers were extracted and homogenized. RNA was obtained and used to analyze the gene expression profile induced by the different insulin modifications. Around 500 genes were upregulated or downregulated by the two insulin derivatives as compared with saline-treated mice (Figure 3A). Albulin and Insulin-Apo profiles were highly similar and only differed in the gene expression of 88 genes. Analysis using Ingenuity software highlighted several genes involved in lipid metabolism that were activated by Albulin but not by Insulin-Apo (Figure 3A). We validated four genes by real-time PCR introducing insulin glargine as an additional control group. Cyp7A1 was used as a marker of insulin activity in the liver as Cyp7A1 has been reported to be repressed by insulin (Park and Pak, 2011). Insulin glargine, Albulin and Insulin-Apo were able to decrease the expression of Cyp7A1. Three genes involved in lipid metabolism and differentially regulated by both insulin modifications were validated: fatty acid synthase (FASN), ATP citrate lyase (ACLY), and acetyl-CoA carboxylase (ACACA). Albulin and insulin glargine increased the expression of the three genes but not by Insulin-Apo (Figure 3B).

### Long-Term Treatment in the db/db Mouse Model

**FIGURE 4:**
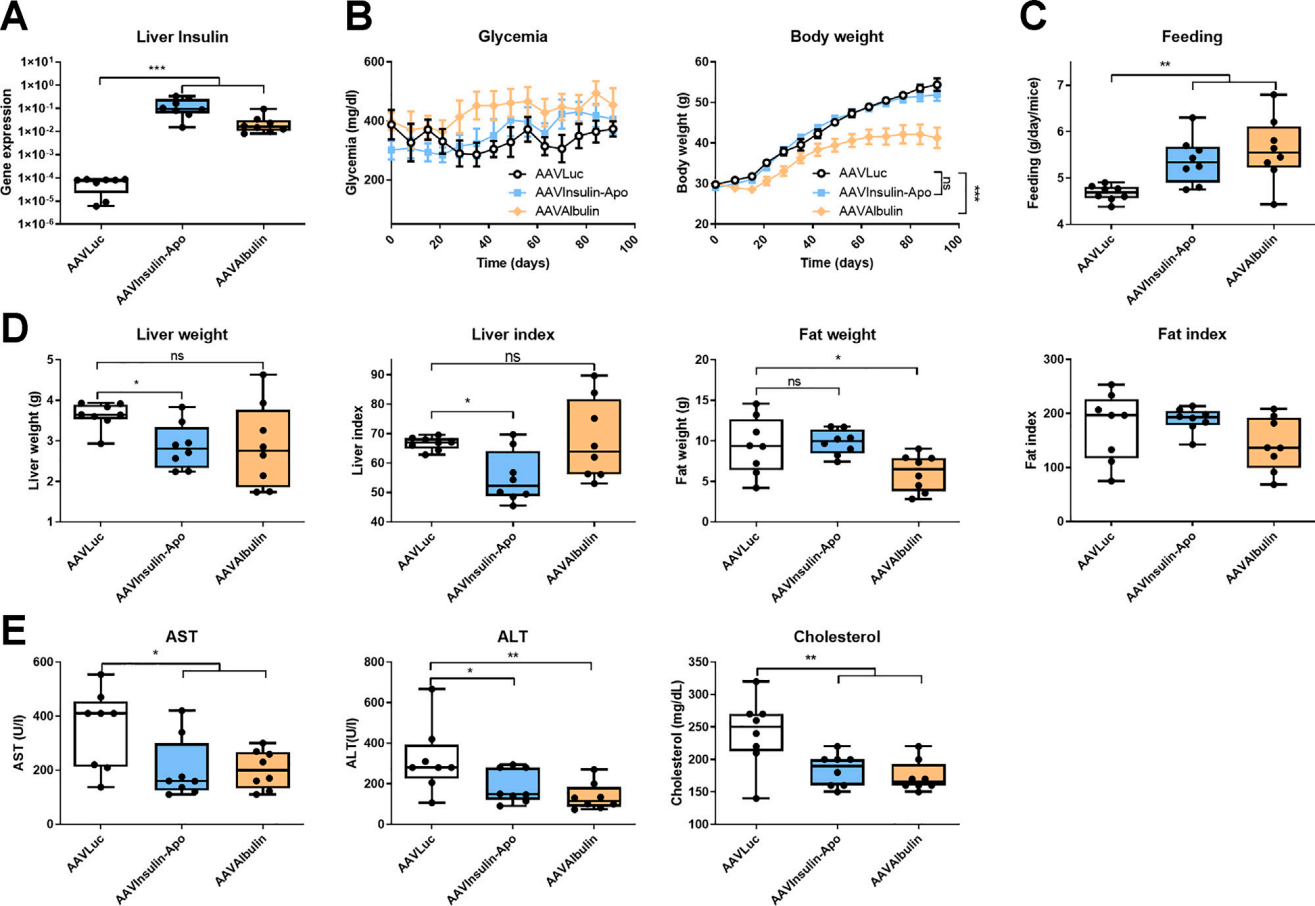
Effect of AAV-mediated expression of insulin derivatives in db/db mice. 10 weeks-old female db/db mice were treated with 5 × 10^12^ viral genomes AAV encoding luciferase (AAVLuc), AAV encoding Insulin-Apo (AAVInsulin-Apo) or AAV encoding albulin (AAVAlbulin). Mice were followed for 3 months and sacrificed at the end of the study. **(A)** Human insulin mRNA levels in the liver 3 months post-injection. **(B)** Evolution of the glycemia and body weight during the study period. **(C)** Average food intake during the study period. **(D)** Liver weight, liver index, fat weight, and fat index 3 months post-injection. **(E)** Serum transaminases (AST and ALT) and total serum cholesterol 3 months post-injection. n = 8 *p < 0.05, **p < 0.01, ***p < 0.001.

**FIGURE 5:**
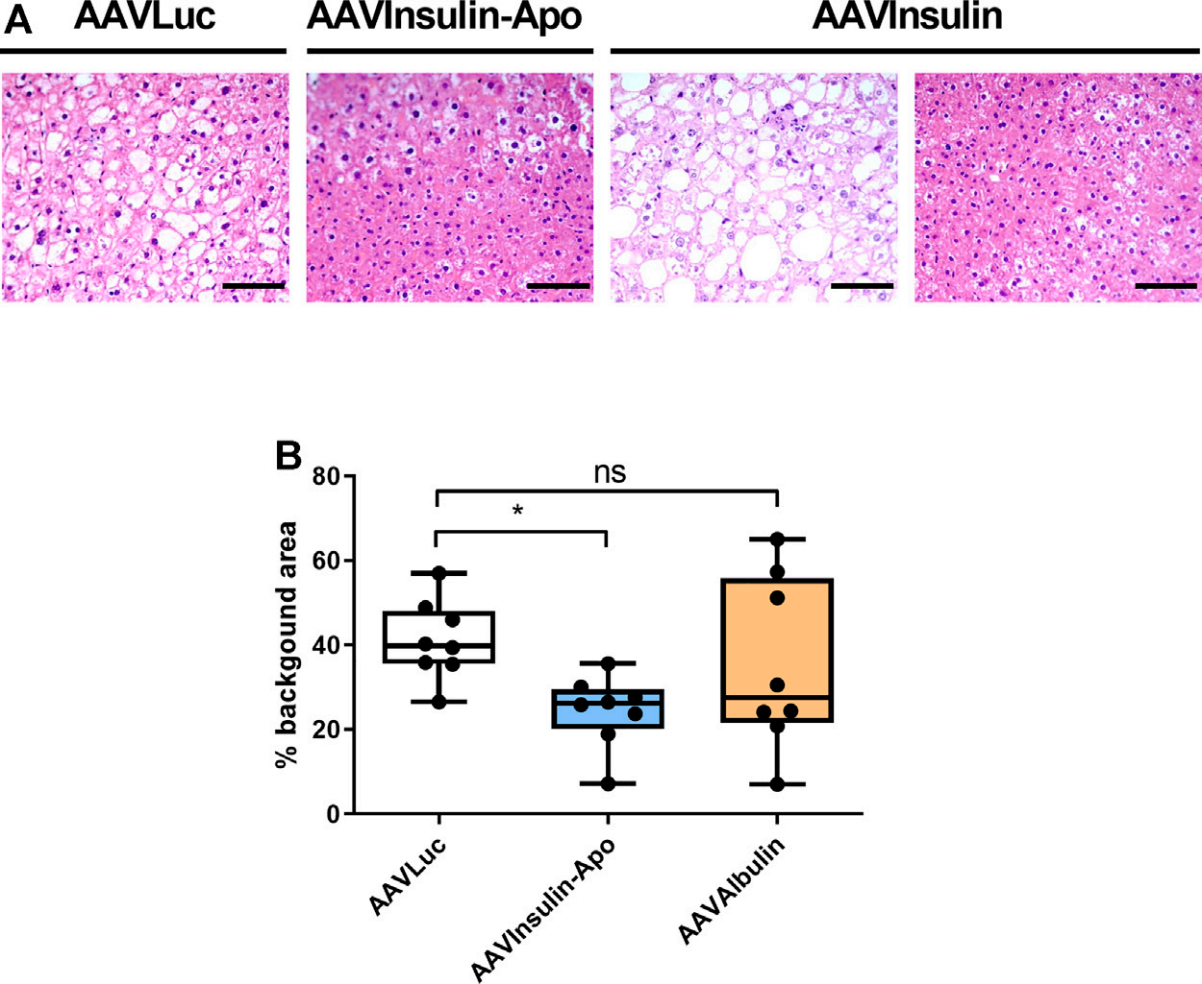
Liver histology 3 months after AAVs injection in db/db mice. **(A)** Hematoxylin-eosin staining of livers from female 10 weeks-old db/db mice treated with 5 × 10^12^ viral genomes AAV encoding luciferase (AAVLuc), AAV encoding Insulin-Apo (AAVInsulin-Apo) or AAV encoding albulin (AAVAlbulin). Scale bars, 50 µm. **(B)** Quantification of the background area in the liver. n = 8 *p < 0.05.

As Insulin-Apo seems to have a different activity profile with respect to liver lipid metabolism, we treated 10 weeks-old female db/db mice, which present an evident overweight and fat deposits in the liver (Bahary et al., 1990). To achieve long-term exposure, we used recombinant adeno-associated vectors (AAV) to express the fusion protein for three months. To allow expression in most of the hepatocytes, we used high doses of the vectors. The *in vivo* activity of the AAV plasmids was evaluated in C57BL/6J mice after hydrodynamic injection of the plasmid (Liu et al., 1999). Both plasmids encoding Insulin-Apo and Albulin were able to decrease the glycemia (Supplementary Figure S2). At the end of the experiment, plasmatic levels of insulin or albumin after administration of the AAV vectors could not be detected, but transgenic mRNAs were present in the liver (Figure 4A). In line with the lack of detection of the insulin derivatives in the plasma, we did not detect glycemia decrease in comparison to a control group treated with an AAV encoding luciferase (AAVLuc). Surprisingly, we detected a marked decrease in body weight in mice treated with the AAV encoding Albulin (AAVAlbulin) but not in the group treated with Insulin-Apo (AAVInsulin-Apo) (Figure 4B). Interestingly, the food intake increased in both AAVInsulin-Apo and AAVAlbulin (Figure 4C). Weight of fat deposits correlated with the body weight in AAVAlbulin treated animals (Figure 4D). In contrast, liver index (the ratio between the liver weight and the bodyweight) showed a significant decrease in mice treated with AAVInsulin-Apo as compared to liver index in the groups treated with AAVLuc or AAVAlbulin. The latter group exhibits high variability in the size of organs, reflecting the positive effect of the bodyweight reduction and the negative effect of liver expression of AAVAlbulin (Figure 4D). Liver expressions of both insulin fusion proteins were able to reduce the serum levels of AST and ALT transaminases and of total cholesterol (Figure 4E). Hematoxylin and eosin staining of liver sections showed large hepatocytes due to the accumulation of fat in control mice. Mice treated with AAVInsulin-Apo presented almost normal liver histology while in the group of AAVAlbulin, we could find both mice with increased steatotic liver alterations and mice with almost physiologic liver architecture (Figure 5A). Due to this variability, the only group with a significant normalization of liver histology was the AAVInsulin-Apo (Figure 5B).

### Therapeutic Effects of Repeated Administration of Recombinant Proteins in the db/db Mouse Model

**FIGURE 6:**
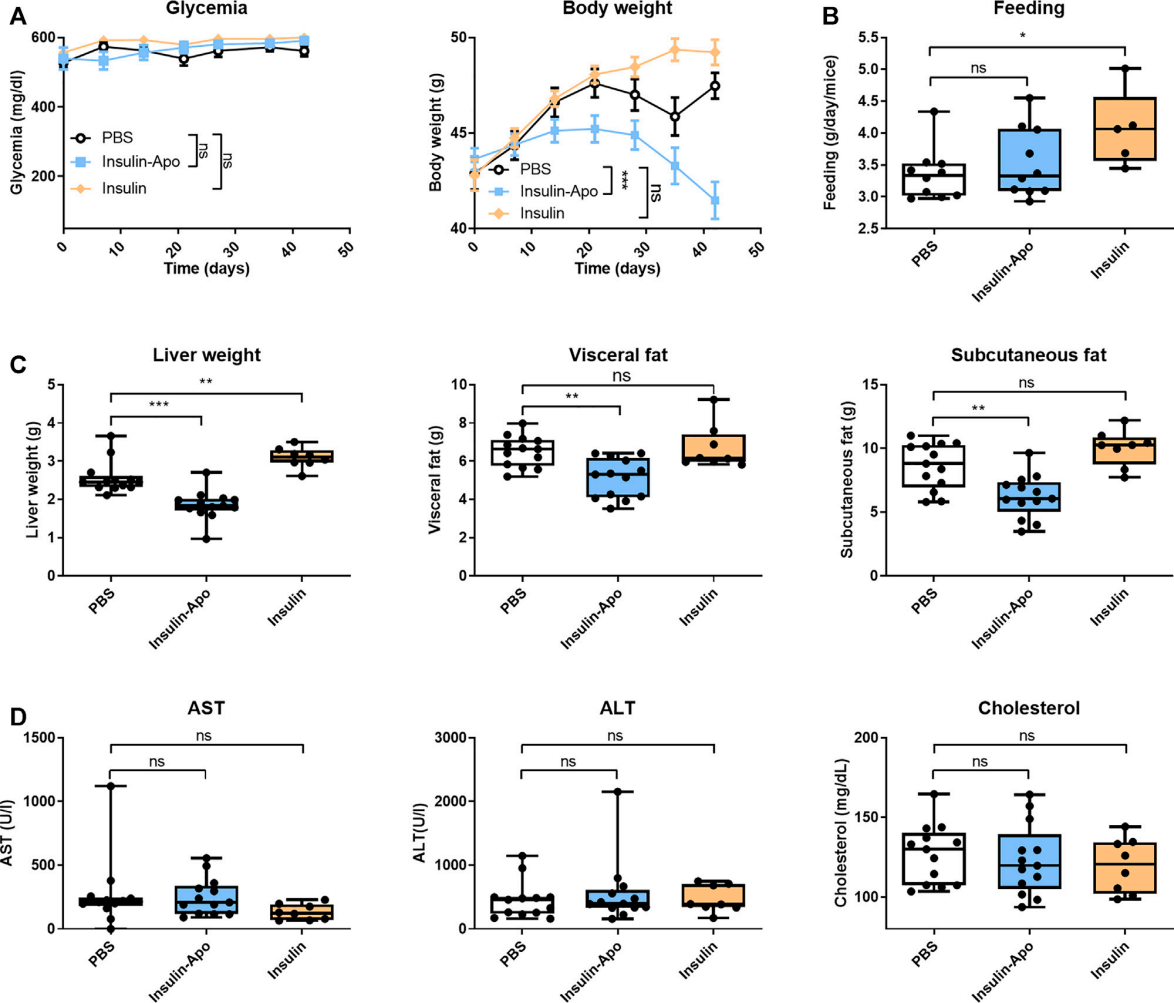
Effect of long term treatment with insulin derivatives in db/db mice. Male 10 weeks-old db/db mice were treated daily for six weeks with PBS, 5 U/kg human insulin s.c., or 26 nmol/kg Insulin-Apo s.c. Mice were sacrificed at the end of the study. **(A)** Evolution of the glycemia and body weight during the study period. **(B)** Average food intake during the study period. **(C)** Liver weight, visceral fat weight, and subcutaneous fat weight after treatment for 6 weeks **(D)** Serum transaminases (AST and ALT) and total serum cholesterol 6 weeks after starting the treatment. n = 13 for PBS and Insulin-Apo groups and n = 8 for the insulin-treated group. *p < 0.05, **p < 0.01, ***p < 0.001.

**FIGURE 7:**
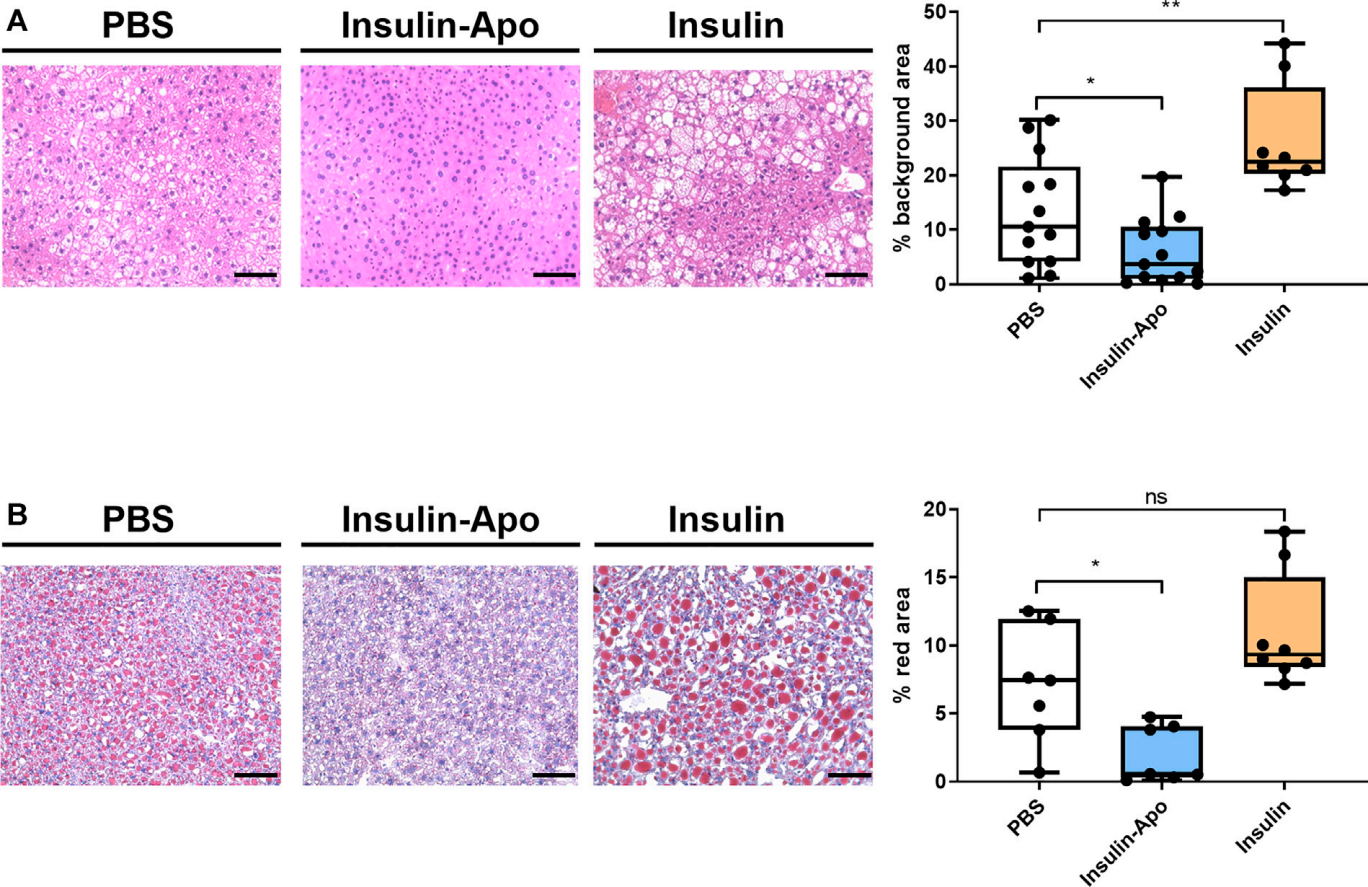
Liver histology 6 weeks after daily s.c. administration of insulin derivatives in db/db mice. **(A)** Representative images of hematoxylin-eosin staining of livers from male 10 weeks-old db/db mice treated daily for 6 weeks with PBS, 5 U/kg human insulin s.c. or 26 nmol/kg Insulin-Apo s.c. and quantification of the background area. Scale bars, 50 μm n = 13 for PBS and Insulin-Apo groups and n = 8 for the insulin-treated group. ***p < 0.001. **(B)** Representative images of Oil red O staining of livers from 10 weeks-old db/db mice treated daily for six weeks with PBS, 5 U/kg human insulin s.c. or 26 nmol/kg Insulin-Apo s.c. and quantification of the red area. Scale bars, 50 μm n = 7 for PBS and Insulin-Apo groups and n = 8 for the insulin-treated group. ***p < 0.001.

**FIGURE 8:**
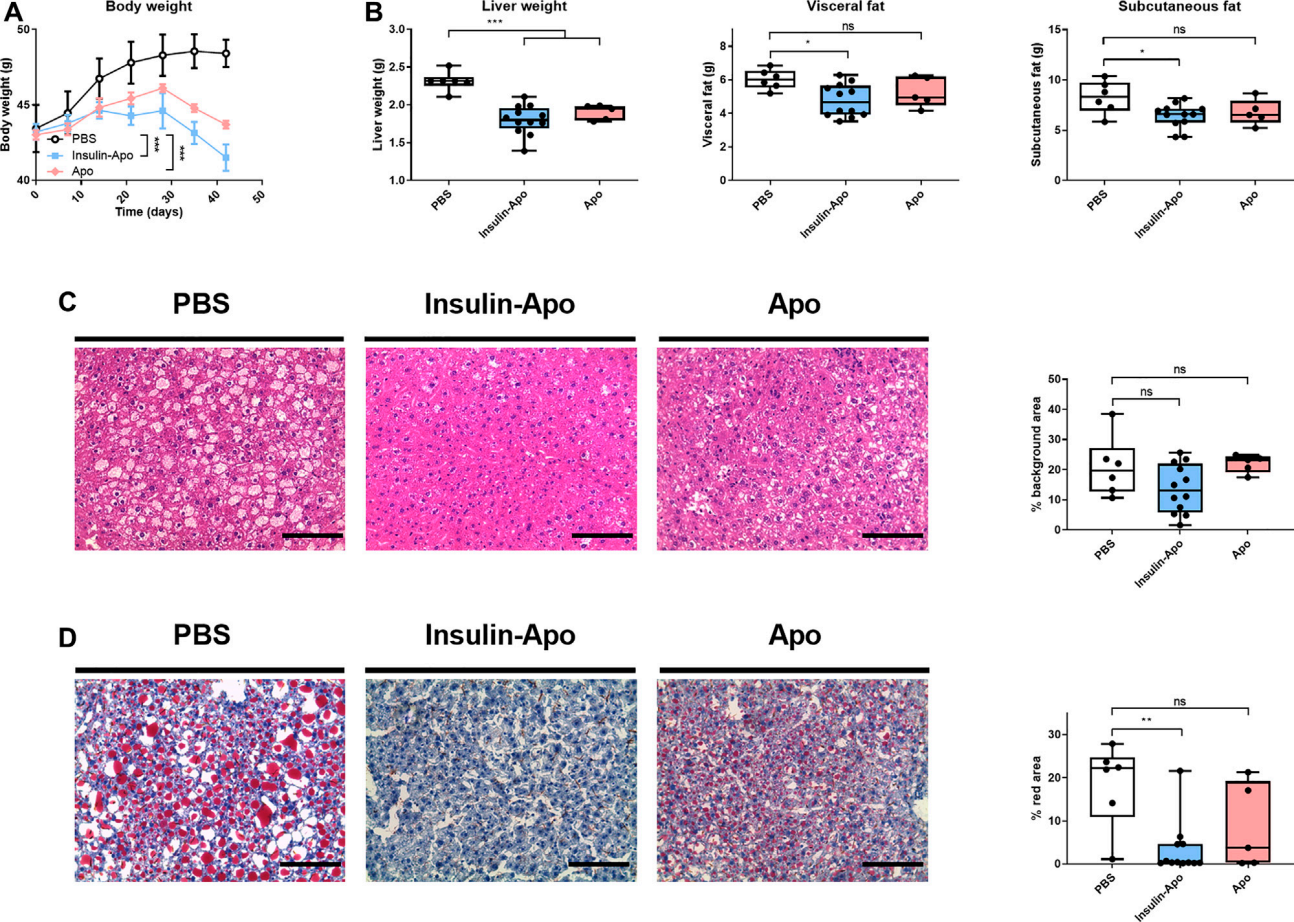
Effect of long term treatment of apolipoprotein A-I alone or fused to insulin in db/db mice. Male 10 weeks-old db/db mice were treated daily for six weeks with PBS, 26 nmol/kg human apolipoprotein A-I s.c., or 26 nmol/kg Insulin-Apo s.c. Mice were sacrificed at the end of the study. **(A)** Evolution of body weight during the study period. **(B)** Liver weight, visceral fat weight, and subcutaneous fat weight after treatment for 6 weeks **(C)** Representative images of hematoxylin-eosin staining of livers after treatment for 6 weeks and quantification of the background area. Scale bars, 50 µm. **(D)** Representative images of Oil red O staining of livers after treatment for 6 weeks and quantification of the red area. Scale bars, 50 μm n = 6 for PBS group, n = 12 for Insulin-Apo-treated group and n = 5 for Apo-treated group *p < 0.05, **p < 0.01, ***p < 0.001.

To confirm these findings, we treated male db/db mice with human insulin or Insulin-Apo with daily subcutaneous administrations for 6 weeks. No antibodies were detected at any time point against the mouse Insulin-Apo (data not shown). The insulin or Insulin-Apo did not have an impact on the baseline levels of serum glucose in these diabetic mice (Figure 6A). Interestingly, Insulin-Apo induced a significant reduction of body weight as compared to insulin-treated animals (Figure 6A) that does not correlate with a reduced feeding (Figure 6B). When mice were sacrificed, a significant decrease in liver weight as well as in fat deposits was observed in animals treated with Insulin-Apo (Figure 6C). No detrimental effects of the treatment with Insulin-Apo were detected, analyzing transaminase levels or circulating cholesterol (Figure 6D). The effects on liver weight correlated with an improvement of liver histology in those animals treated with Insulin-Apo while administration with insulin made the liver disease worse, as shown by hematoxylin and eosin staining (Figure 7A) and by the lipid-specific staining Oil Red O (Figure 7B). To determine the contribution of the apolipoprotein A-I moiety to the overall effect of the fusion protein, db/db mice were treated with apolipoprotein A-I or Insulin-Apo daily for 6 weeks. Although apolipoprotein A-I exerted a potent effect on body weight, the fusion proteins outperformed the unconjugated apolipoprotein A-I (Figure 8A). Moreover, significant differences were detected when liver weight and subcutaneous fatty deposits in the group treated with saline were compared to the group treated with Insulin-Apo. However, no significant differences were observed in mice treated with apolipoprotein A-I alone (Figure 8B). Insulin-Apo was also more efficient in normalizing liver histology (Figure 8C), and a significant reduction in the fat accumulation in the liver was achieved (Figure 8D).

## Discussion

Here, we have analyzed the pharmacokinetics and pharmacodynamics of the fusion protein of apolipoprotein A-I and a single chain insulin. This fusion protein was able to promote the glucose uptake and the phosphorylation of insulin receptor and AKT. However, the activity of the recombinant protein was 10-fold lower than the activity of insulin. Similar reductions of the *in vitro* activity has been reported for other insulin modifications such as albumin fusion to insulin (Shechter et al., 2005), immunoglobulin Fc domain fusions (Wronkowitz et al., 2017), insulin detemir (Sorensen et al., 2010), but this pharmacodynamic disadvantage can be compensated with an improved pharmacokinetic profile. In the case of Insulin-Apo, the pharmacokinetic data are compatible with a long-lasting insulin formulation that could be administered once daily. The hypoglycemic effect after an intravenous administration showed a slow onset, a profile observed with the fusion protein of proinsulin-transferrin (Wang et al., 2014), and may represent a general property of liver targeted insulins.

In order to analyze whether the novel insulin derivative could exert any differential effects in the liver, we analyzed the phosphorylation of the insulin receptor in the liver, fat, and muscle. Insulin-Apo markedly increased the phosphorylation of the insulin receptor in the liver 6 h after administration while insulin phosphorylated the receptor in muscle and fat but not in the liver. To further analyze the liver activity of Insulin-Apo, we performed microarray analysis 6 h after administration of the long-lasting insulins. A subset of differentially expressed genes predicted that the lipid metabolism was upregulated by the single-chain insulin fused to albumin but not with the fusion between insulin and apolipoprotein A-I. To validate these results in a relevant animal model, we treated obese diabetic female db/db mice that already presented accumulation of lipids in the liver (Bahary et al., 1990). A single dose of an AAV vector allowed us to treat these mice for three months and analyze the long-term evolution of the disease (Paneda et al., 2009). The expression was low as reflected by the lack of glycemia reduction. Surprisingly, a significant decrease in body weight was observed in mice treated with the AAVAlbulin. This unexpected result may be due to the sustained release of low doses of insulin, pharmacokinetics that might be replicated by low doses of the novel ultra-long lasting insulins (Gough et al., 2012). Liver index and liver histology reflected that liver steatosis were promoted in half of the animals treated with AAVAlbulin. In this group, a balance between the positive effect of weight loss and the negative effect of the direct activity of insulin in an insulin-resistant liver determines the final outcome. The group treated with AAVInsulin-Apo presented normalization of liver histology with a significantly reduced liver index and a significant decrease in the vacuoles present in hepatocytes. To validate these findings in a clinically relevant drug formulation, we produced large amounts of the recombinant fusion protein and the long-term activity on diabetes, obesity and steatosis was determined in db/db mice. We used male mice for these experiments since they tend to be more susceptible to NAFLD development than female mice (Matsushita et al., 2017). Daily subcutaneous administration of the recombinant protein reduced the body weight and improved liver histology. In contrast, administration of human insulin worsened both obesity and steatosis. Administration of apolipoprotein A-I alone also has a significant impact on body weight as has been previously reported (Ruan et al., 2011) and on liver weight, indicating that the apolipoprotein A-I moiety is the major driver of the effects on body weight and steatosis. However, the fusion protein outperformed the apolipoprotein A-I effects in all analyzed parameters.

In conclusion, insulin fused to apolipoprotein A-I is a novel long-lasting insulin analog with preferential activity in the liver. Due to the effects of apolipoprotein A-I on body weight and steatosis, it may be of interest for the treatment of diabetic patients, especially with NAFLD.

## Data Availability

The raw data generated for this article can be accessed from NCBI using the accession number GSE160105.
