# Supplementary material for: Insulin Fused to Apolipoprotein A-I Reduces Body Weight and Steatosis in DB/DB Mice
Source: Front Pharmacol. 2021 Feb 19;11:591293. doi: 10.3389/fphar.2020.591293 (PMC7934061; doi:10.3389/fphar.2020.591293)
Supplement: Supplementary file 1 [file datasheet1.pdf]

Supplementary Material

1 Supplementary Figures

A

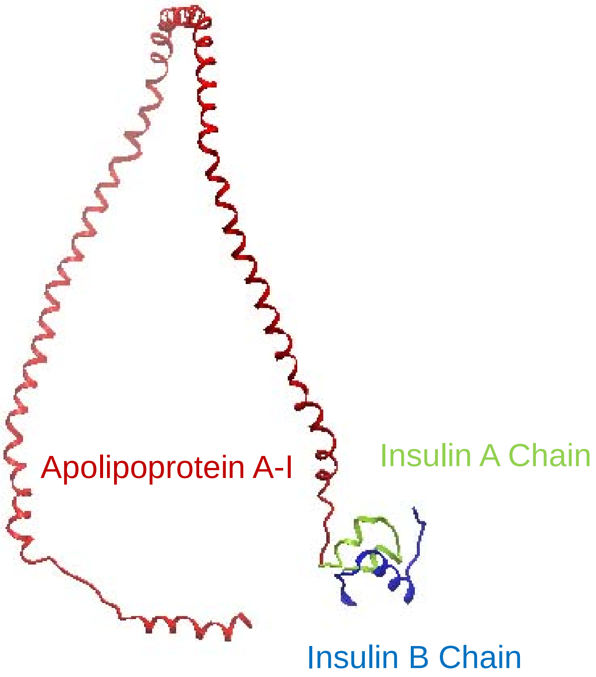

B

```
1   MFVNQHLCGS HLVEALYLVC GERGFFYTPK TQRGGGGGQR GIVEQCCTSI CSLYQLENYC

61  NGAPDEPPQS PWDRVKDLAT VYVDVLKDSG RDYVSQFEGS ALGKQLNLKL LDNWDSVTST

121 FSKLREQLGP VTQEFWDNLE KETEGLRQEM SKDLEEVKAK VQPYLDDFQK KWQEEMELYR

181 QKVEPLRAEL QEGARQKLHE LQEKLSPLGE EMRDRARAHV DALRTHLAPY SDELRQRLAA

241 RLEALKENGG ARLAEYHAKA TEHLSTLSEK AKPALEDLRQ GLLPVLESFK VSFLSALEEY

301 TKKLNTQHHH HHH
```

Supplementary Figure 1. Fusion protein of a single chain insulin and apolipoprotein A-I. (A) Representation of the apolipoprotein A-I (red) fused to the insulin A chain (green) and the insulin B chain (blue). (B) Aminoacid sequence of the fusion protein using the previous color code.

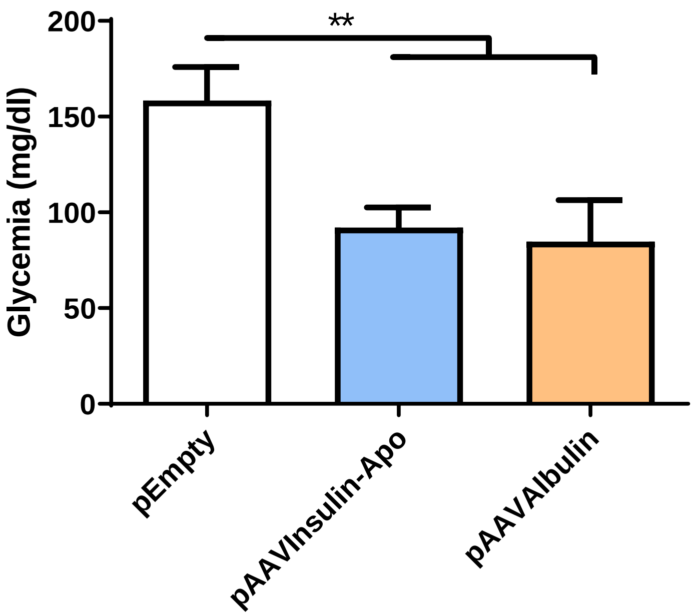

Supplementary Figure 2. In vivo activity of AAV constructs. C57BL/6 mice were treated with a hydrodynamic injection of a control plasmid (pEmpty), a plasmid harboring the genome of the AAV encoding insulin-apo (pAAVInsulin-Apo) or a plasmid harboring the genome of the AAV encoding albulin (pAAVAlbulin). 24h later, glycemia was analyzed with a glucometer. ** $P<0.01$.

2 Supplementary Tables

Name	Sequence
Insulin-Fw	5’-CTACTGGCCAACCTGCTCTC-3’
Insulin-Rv	5’-CTGGCCACCGCCACC-3’
ACACA-Fw	5’-TTCTAACAGGAGCTGGAGCC-3’
ACACA -Rv	5’-AGTGGGTCACCCCATTGTT-3’
ACLY-Fv	5’-TCTTTGTGCTGGGAAGGAGT-3’
ACLY -Rv	5’-CATCCCACGGATGACGATAC-3’
FASN-Fw	5’-AACTCCAAGGACACAGTCACCAT-3’
FASN-Rv	5’-CAGCTGCTCCACGAACTCAA-3’
CYP7A1-Fw	5’-GCTGTGGTAGTGAGCTGTTGCA-3’
CYP7A1-Rv	5’-CACAGCCCAGGTATGGAATCA-3’
RPLP0-Fw	5’-AACATCTCCCCCTTCTCCTT-3’
RPLP0-Rv	5’-GAAGGCCTTGACCTTTTCAG-3’

Supplementary Table 1. List of primers.
